# A Deep Learning Model for Markerless Pose Estimation Based on Keypoint Augmentation: What Factors Influence Errors in Biomechanical Applications?

**DOI:** 10.3390/s24061923

**Published:** 2024-03-17

**Authors:** Ana V. Ruescas-Nicolau, Enrique Medina-Ripoll, Helios de Rosario, Joaquín Sanchiz Navarro, Eduardo Parrilla, María Carmen Juan Lizandra

**Affiliations:** 1 Instituto de Biomecánica-IBV, Universitat Politècnica de València, Edifici 9C, Camí de Vera s/n, 46022 Valencia, Spain; enrique.medina@ibv.org (E.M.-R.); helios.derosario@ibv.org (H.d.R.); joaquin.sanchiz@ibv.org (J.S.N.); eduardo.parrilla@ibv.org (E.P.); 2 Instituto Universitario de Automática e Informática Industrial, Universitat Politècnica de València, Edifici 1F, Camí de Vera, s/n, 46022 Valencia, Spain; mcarmen@dsic.upv.es

**Keywords:** markerless, deep learning, anatomical landmark, human pose estimation, biomechanics, keypoint augmentation

## Abstract

In biomechanics, movement is typically recorded by tracking the trajectories of anatomical landmarks previously marked using passive instrumentation, which entails several inconveniences. To overcome these disadvantages, researchers are exploring different markerless methods, such as pose estimation networks, to capture movement with equivalent accuracy to marker-based photogrammetry. However, pose estimation models usually only provide joint centers, which are incomplete data for calculating joint angles in all anatomical axes. Recently, marker augmentation models based on deep learning have emerged. These models transform pose estimation data into complete anatomical data. Building on this concept, this study presents three marker augmentation models of varying complexity that were compared to a photogrammetry system. The errors in anatomical landmark positions and the derived joint angles were calculated, and a statistical analysis of the errors was performed to identify the factors that most influence their magnitude. The proposed Transformer model improved upon the errors reported in the literature, yielding position errors of less than 1.5 cm for anatomical landmarks and 4.4 degrees for all seven movements evaluated. Anthropometric data did not influence the errors, while anatomical landmarks and movement influenced position errors, and model, rotation axis, and movement influenced joint angle errors.

## 1. Introduction

In the clinical setting, the analysis of human movement is important for understanding and managing diseases of the musculoskeletal system. For example, the analysis of the mobility of the cervical spine can facilitate the treatment of conditions such as low back pain or cervical pain [1,2,3]. Similarly, gait analysis allows the diagnosis and subsequent planning and evaluation of treatment of neuromusculoskeletal pathology [4,5].

Marker-based photogrammetry motion capture (MoCap) is considered the gold standard for the analysis of human movement, demonstrating the ability to provide comprehensive, accurate, robust, and reproducible data. However, despite being the gold standard, its daily clinical practice application is constrained by certain drawbacks associated with this technique.

The main reason discouraging movement assessment using marker-based photogrammetry systems is the high cost, both financially and in terms of time, with instrumentation of the participants being one of the main problems [6]. Normally, it is necessary to identify a series of anatomical landmarks and to fix reflective markers in order to calculate the anatomical axes and kinematics of each joint [7,8]. This operation requires highly qualified personnel and is very time consuming. In addition, differences in marker placement directly affect the reproducibility and accuracy of the measurements [9,10,11].

In order to overcome these limitations, commercial markerless motion capture systems have been developed in recent years, such as Theia3D (www.theiamarkerless.ca, accessed on 18 January 2024), Captury (www.captury.com, accessed on 18 January 2024), Fast Move AI 3D (www.fastmove.cn, accessed on 18 January 2024), Kinatrax Motion Capture (www.kinatrax.com, accessed on 18 January 2024), and OpenCap (www.opencap.ai, accessed on 18 January 2024). Many of these systems accurately estimate kinematics in the principal plane [12] but tend to have larger errors in the measurement of secondary angles. Additionally, many of these approaches require multiple cameras, specific software, and specialized computing resources [13,14].

An alternative to vision systems is inertial systems. Inertial measurement units are portable and can accurately estimate kinematics [15,16]. However, commercial sensors still present similar problems: long instrumentation time, the use of proprietary algorithms, and the low-cost sensors are often prone to errors of misalignment, orthogonality, and offset, which require correction methods to achieve accurate position and orientation measurements [17].

In recent years, new methods of human pose detection based on images have been developed [18,19,20,21,22,23]. Thus, by triangulating the keypoints identified by these pose estimation algorithms in various videos, their 3D positions can be obtained [24,25].

These pose detection methods are usually trained using image datasets where the labeling (localization of keypoints) was performed manually, which may result in inconsistencies and low accuracy [26].

There are datasets in which the images were labeled with reliable and accurate marker-based MoCap [27,28,29,30] but the collected images feature subjects that are altered by the presence of external elements, such as reflective spheres. This alteration prevents their use for training general computational models. In such cases, the detector would be at risk of learning to localize the presence of external elements rather than the anatomical location of keypoints, especially when they are arranged in a fixed pattern [31].

According to [26], open-source pose estimation algorithms were never designed for biomechanical applications. In fact, they usually provide the centers of the main joints but not the key anatomical landmarks needed to fully characterize the translations and rotations of all body segments. There have been different ways to improve the accuracy of pose estimation methods so that they can be used in the analysis of human motion from a biomechanical point of view.

The most widespread solutions involved generating proprietary image datasets for training the pose estimator. For example, the markerless Theia3D system was trained with 500k images in the wild labeled by a group of expert taggers [32,33,34]. Other solutions were based on generating image datasets of specific movements, such as [35,36], who created ENSAM and its extended version in order to fine-tune the pose estimator for gait applications. Another example is presented in work [30], where the GPJATK dataset is introduced, specifically created for markerless 3D motion tracking and 3D gait recognition.

Another approach involves feeding a biomechanical model with the 3D landmarks detected in synchronized videos. For example, in [37], the 3D reconstructed keypoints were used to drive the motion of a constrained rigid-body kinematic model in OpenSim, from which the position and orientation of each segment could be obtained [38].

A recent approach is based on augmenting the keypoints of a pose estimator in images with anatomical markers [25]. In this approach, the authors proposed using a marker augmenter, a long short-term memory (LSTM) network, that estimates a set of anatomical landmarks from the reconstructed keypoints detected in videos. Two LSTM networks were trained for an arm and full body, with 3D video keypoints, weight and height, and 3D anatomical landmarks from 108 h of motion captures processed in OpenSim 4.3.

We were inspired by the latter strategy, as it leverages pose estimation technology from images to solve a different aspect of the problem, and it is also suitable for application to a wide range of movements. However, we wondered if this strategy could be applied with any marker augmentation model and what other factors would influence its performance. Therefore, we present a comparative study in which we assess three marker augmenters for lower limbs across eight different poses and movements with 14 subjects, using the MediaPipe Pose Landmarker model as the pose detector [23]. We utilized the “Human tracking dataset of 3D anatomical landmarks and pose keypoints” [39], which is a collection of 3D reconstructed video keypoints associated with 3D anatomical landmarks extracted from 567 sequences of 71 participants in A-pose and performing seven movements. This dataset was designed for biomechanical applications and is freely available for the research community.

This study had a dual purpose. The first was to assess the performance of the different proposed marker augmentation models. The second was aimed at determining the factors that most influence anatomical landmark estimation and joint angles errors. The factors we considered included the type of marker augmentation model, the movement, the anatomical landmark, the joint, the axis, and the anthropometric characteristics of the subject (height, weight, and sex).

The following sections present the characteristics of the data used in this work, the architectures of the marker augmentation models, and the results of the error evaluation and statistical analysis. Subsequently, the obtained results are discussed, and the conclusions of the work are presented.

## 2. Materials and Methods

In this section, we provide a description of the dataset used in this work, elaborate on the procedure and details of the proposed marker augmentation models, discuss the metrics employed for evaluation, and present the statistical analysis of the errors.

### 2.1. Dataset

A portion of the dataset “Human tracking dataset of 3D anatomical landmarks and pose keypoints” [39] has been used. Section 2.1.1 briefly introduces the workflow followed to obtain the whole dataset. Section 2.1.2 specifies the portion employed and its use in training the models.

#### 2.1.1. Dataset Generation Overview

**Figure 1:**
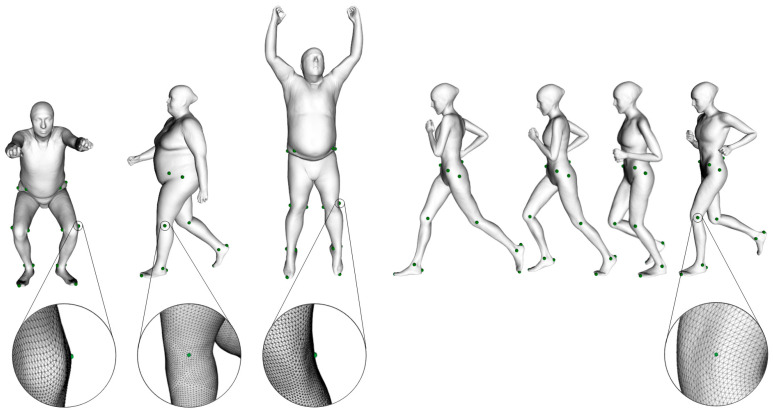
Vertices that correspond to anatomical landmarks on homologous meshes showing the vertex-to-vertex correspondence between subjects (**left**) and throughout the sequence (**right**). The same landmarks correspond to the same vertex ID in all the meshes. Details on the left Femoral Lateral Epicondyle are shown.

The subjects who participated in the dataset collection were scanned with Move4D (www.move4d.net, accessed on 31 January 2024) [40], which is composed of a photogrammetry-based 3D temporal scanner (or 4D scanner) and a processing software. It generates a series of rigged and textured meshes, referred to as homologous meshes, which maintain a consistent vertex-to-vertex correspondence between subjects and through the movement sequence as shown in Figure 1.

Move4D was validated as a good alternative to marker-based MoCap using photogrammetry [41] and its homologous mesh topology allows for considering a set of fixed vertices as representative of anatomical landmark locations [42].

The subjects were scanned at high resolution (49,530 vertices per mesh and a texture image size of 4 megapixels) performing a calibration A-pose and seven movements: gait (60 fps), running (60 fps), squats (30 fps), jumping jacks (j-jacks, 30 fps), counter movement jump (jump, 60 fps), forward jump (f-jump, 30 fps), and turn jump (t-jump, 30 fps). During the sessions, the participants wore tight garments and were asked to remove any clothing or wearable that impeded or distorted their body shape.

The result of processing each scan in each timeframe was a mesh with a realistic texture. These meshes served, on the one hand, to render virtual images from 48 different points of view, and on the other hand, to export the position of the vertices representing the anatomical landmarks, our ground truth. The 3D keypoints were obtained by applying linear triangulation to the 2D keypoints estimated in the virtual images using MediaPipe.

Further details and the complete followed procedure for generating the whole dataset are described in [43], and the data are available in [39].

#### 2.1.2. Description of the Data Used in Training and Validation

As mentioned in the previous section, the whole dataset contains 3D keypoints obtained from two different procedures. The first corresponds to the 3D positions triangulated from 2D keypoints estimated by the MediaPipe pose network from various points of view. The second corresponds to the 3D anatomical landmarks extracted from the homologous mesh for the same subject and timeframe.

**Figure 2:**
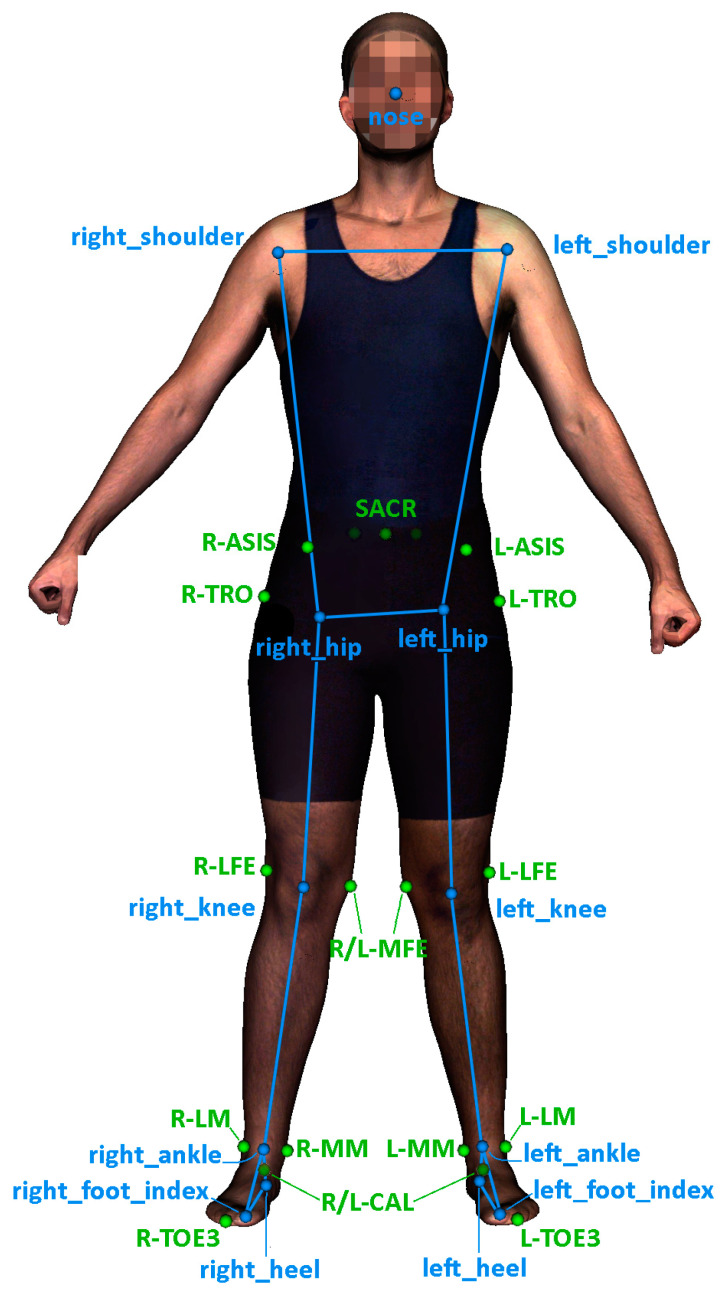
Selected keypoints estimated from MediaPipe pose network (blue) and 3D anatomical landmarks (green) extracted from a homologous mesh.

From the whole dataset, we specifically utilized data related to the lower limbs. In particular, we utilized 13 out of the 33 MediaPipe keypoints and 18 out of the 53 available anatomical landmarks. Figure 2 illustrates the selection of keypoints used in this work.

Anthropometric data, including weight, height, and sex, were used as inputs in all the models.

The dataset was randomly split into two subsets: the training set, composed of 57 subjects, and the test set, composed of 14 subjects. Table 1 shows the anthropometric characteristics of each subset. The code for the subjects in the test subset used in this work is detailed in Appendix A.

### 2.2. Data Pre-Processing

We first substituted R-PSIS and L-PSIS with a new anatomical landmark, SACR, defined as SACR = (R-PSIS + L-PSIS)/2. We carried this out following the recommendation of the International Society of Biomechanics (ISB) to calculate the reference framework for the pelvis, which allows for the use of the midpoint between R-PSIS and L-PSIS [7].

The position of the 3D keypoints and anatomical landmarks was expressed with respect to the midpoint of the hip keypoints and was also scaled by the subject’s height. Finally, min–max normalization was applied to height and weight inputs, considering their value ranges in the whole dataset.

Data augmentation was performed on the training subset sequences by applying multiple rotations around the vertical axis.

### 2.3. Marker Augmentation Models

To investigate the effect of the type of marker augmentation model on the estimation errors of position and joint angles, we utilized three marker augmentation models. These models estimate the anatomical landmarks required for calculating the joint kinematics of the lower limb. We selected these models to represent neural networks of varying complexity, enabling us to examine their suitability for marker augmentation purposes. The models presented are listed below:

- MLP: a multilayer perceptron with a rectified linear unit activation function (ReLU). This model is less accurate but very lightweight, allowing its implementation on devices with low resources such as mobile phones or other low-cost devices.
- LSTM: an adaptation of the long-short term memory neural network used in OpenCap for the full body [25]. It uses temporal but not spatial information.
- Transformer [44]: designed for a comprehensive understanding of the problem, capturing long-range dependences in the global context. This model improves upon the previous one by incorporating spatial information. Transformers have recently been used successfully in different problems. It is more resource intensive but more accurate.

The same numbers of inputs and outputs were set for all the models. They had 42 inputs: sex, weight, height, the 3 × 13 coordinates of the 3D keypoints, and 51 outputs, which were the 3 × 17 coordinates of the selected anatomical landmarks (Figure 2). The architectures and training parameters of the models are described in the following subsections. The development and the training of the models were carried out in Python 3.8.10 using Keras 2.7.0 and Tensorflow backend 2.7.0 [45,46].

#### 2.3.1. MLP Model

**Figure 3:**
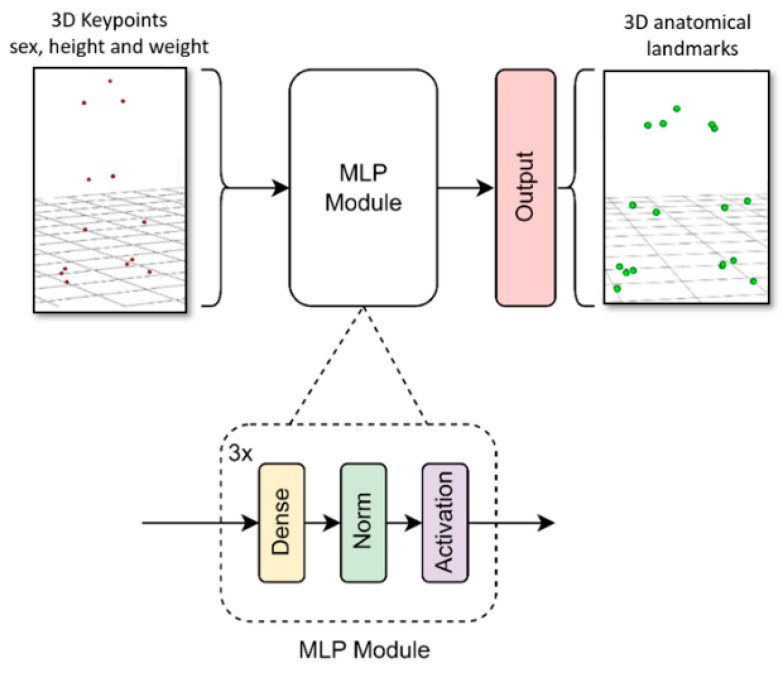
MLP model architecture diagram.

The multilayer perceptron model we developed consisted of several blocks (see Figure 3). Each block comprised a dense layer, followed by a batch normalization layer and an activation layer. Keras Tuner was used to determine the network’s hyperparameters [47]. Specifically, an optimization was performed to determine the number of blocks, the number of units in the dense layer, the activation function (hyperbolic tangent or ReLU), and the learning rate. This process took about one week using an Nvidia GTX 1050 Ti.

The final architecture comprised three blocks. The first one had 256 units in the dense layer with a hyperbolic tangent activation function. The activation functions for the second and third blocks were ReLU, with 128 and 224 units in the dense layer, respectively. The input layer consisted of 42 units (weight, height, sex, and the 3D coordinates of 13 keypoints from a specific frame). The output layer consisted of 51 units (3D coordinates of 17 anatomical points). The learning rate was set to 2 × 10^−5^, the optimizer selected was RMSprop, and the loss function was the mean squared error (MSE). The training process took 100 epochs with batch size of 64 (approximately one hour of training using an Nvidia GTX 1050 Ti).

#### 2.3.2. LSTM Model

**Figure 4:**
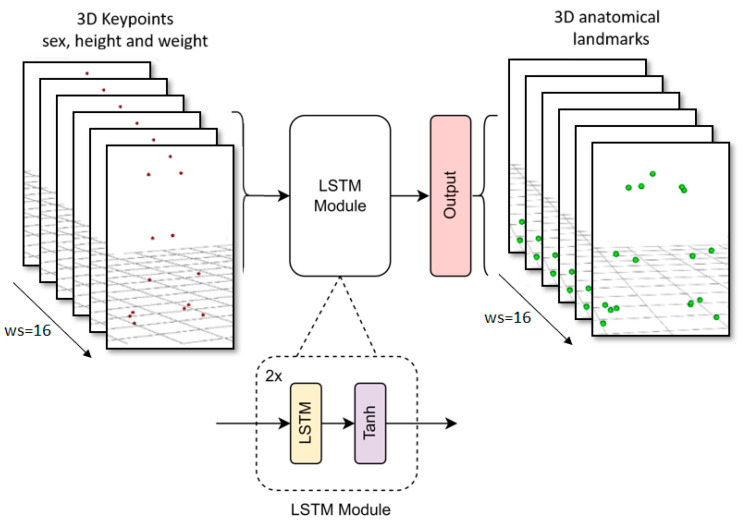
LSTM model architecture diagram.

We adapted the input and output shape of the LSTM body model presented in [25] with a sequence length of 16 frames. This model comprised 2 LSTM layers with 128 units in each layer, followed by a dense layer with linear activation. The learning rate was set to 7 × 10^−5^, the optimizer selected was Adam, and the loss function was mean squared error (MSE), while the other parameters remained at their default values. The training process took 200 epochs with batch size of 32 (approximately twelve hours of training using an Nvidia GTX 1050 Ti). Figure 4 shows a diagram of the proposed LSTM.

#### 2.3.3. Transformer Model

The model’s input consists of a series of 3D keypoints and anthropometric data. This 3D information tensor was initially transposed so that the number of keypoints matched the channel dimension, allowing for linear projection to the Transformer’s hidden dimension using a Conv2D with a kernel size of (1, 1).

**Figure 5:**
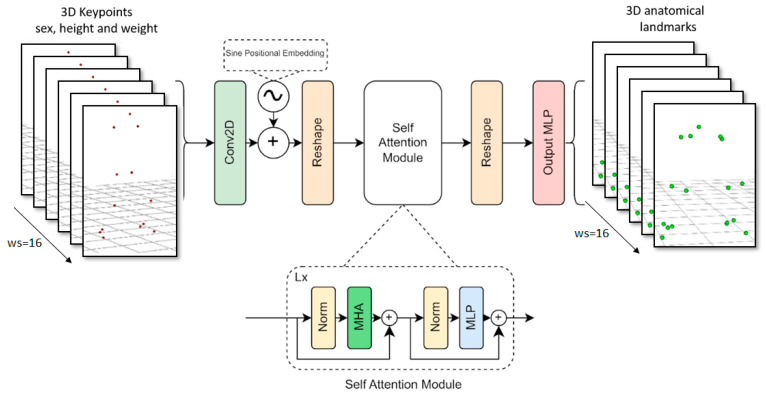
Transformer model architecture diagram.

Next, the sine positional embedding layer added spatial information to the matrix [48], which was then flattened to match the Transformer’s input shape. These projected features were passed through 6 self-attention layers with 14 heads, and then reshaped to match the input of the MLP head. The MLP head outputs the sequence of 3D anatomical landmark time series with a linear activation (see Figure 5).

The learning rate, optimizer hyperparameters, and loss function were the same as those used in the LSTM model. The training process took 300 epochs with batch size of 64 (approximately one day of training using two Nvidia RTX 3090).

### 2.4. Metrics

The Euclidean distance between observed and reconstructed anatomical landmarks, averaged over every movement sequence across all subjects in the test subset, was used to characterize the errors in the estimation of the anatomical landmarks positions, whereas errors in the joint angles were parametrized as the root mean squared error (RMSE), as in [12,25,49,50].

The anatomical axes and angles of the hip, knee, and ankle were calculated according to [7,51] using the positions of the anatomical landmarks. The hip joint center was obtained following the procedure described in [52].

The trochanterion landmarks were used as technical markers on the thighs in the calculations of the joint angles.

### 2.5. Error Analysis

Means and standard deviations of those errors were calculated, and their distributions for different movements, anatomical landmarks, and axes were compared across models. In order to quantify the influence of those factors on the size of the errors, linear mixed models (LMMs) were fitted using the subject as a random factor and the following fixed factors:

- Prediction model, movement, and anatomical landmarks for the prediction of anatomical landmark locations;
- Prediction model, side of the body joint, and rotation axis for the calculation of joint angles.

The LMMs also accounted for possible interactions between the effects of: (a) model and movements for both types of errors; (b) model and anatomical landmarks for errors in anatomical landmark locations; (c) model, joint, and rotation axis for errors in joint angles.

An analysis of variance (ANOVA) was used to compare those LMMs to others that also included the characteristics of the subjects (sex, height, and weight) as fixed factors in order to test whether the errors could be assumed to be independent of the subject’s anthropometry.

The analysis was carried out with the R 4.1.3. software for statistical computing [53], using the packages lmerTest, performance, and phia [54,55,56].

## 3. Results

In this section, we present the errors obtained with each model and the results of the statistical analysis performed. 

### 3.1. Anatomical Landmark Position Errors

The average errors per anatomical landmark and movement fell within the following ranges: [0.77, 3.75] cm for the MLP model, [0.64, 2.74] cm for LSTM model, and [0.55, 2.11] cm for Transformer model.

All three evaluated models estimated the anatomical landmarks located around the pelvis (L/R-ASIS, L/R-TRO, SACR) with the greatest errors (ranging from 1.88 to 2.23 cm), while those around the ankles (L/R-LM, L/R-MM, L/R-CAL) were estimated with the lowest errors (ranging from 0.88 to 1.36 cm).

Regarding the errors per movement, the greatest errors were observed in the running movement, with mean errors of 2.73 cm for the MLP model, 1.84 cm for the LSTM model, and 1.81 cm for the Transformer model. Errors for the A-pose were the smallest, around 1.1 cm for all models, followed by jumps and squats, for which errors were approximately 1.57 for MLP, 1.31 cm for LSTM, and 1.36 cm for Transformer. The complete anatomical landmark position errors are shown in Table 2 (MLP), Table 3 (LSTM), and Table 4 (Transformer).

### 3.2. Joint Angle Errors

The RMSDs per movement and axis were limited within the following ranges: [2.52, 15.35] degrees for the MLP model, [1.78, 9.32] degrees for the LSTM model, and [1.91, 7.13] degrees for the Transformer model. 

While the worst results in the MLP model were found in the ankle angles (over 5.8 degrees), the LSTM and Transformer models obtained the worst results in the hip angles. The knee angles were generally well estimated (under 4.4 degrees), except for the rotation in the MLP model (9.06 degrees). 

All the models obtained their worst results in the running movement (ranging from 4.32 to 9.29 degrees) and the best results in jump and squat movements (ranging from 3.19 to 4.93 degrees).

Table 5, Table 6 and Table 7 show detailed joint angle errors for the MLP, LSTM, and Transformer models, respectively. 

### 3.3. Factors Influencing the Errors

The subjects’ characteristics had no significant influence on the errors (*p* = 0.573 for anatomical landmark errors, *p* = 0.758 for joint angle errors). Table 8 and Table 9 show the results of the ANOVA for the LMM fitted without those characteristics; values of the statistical tests are omitted, since due to the large amount of data points, the null hypothesis (no effect of the factors) would always be rejected even for negligible effect sizes.

The R^2^ values in those tables show that around half of the variance was random error not explained by the considered factors and that the random influence of the subjects (difference between conditional and marginal R^2^) was also small [57]. The sums of squares show that, for anatomical landmark position errors, the effect of the model was smaller than the effects of the movement and the anatomical landmark and that the effects of the interactions were one order of magnitude smaller. For joint angle errors, on the other hand, the model was the greatest source of variation, and there were also important interactions between effects, especially between those of the joint and the rotation axis; the side of the body, however, barely affected the results.

**Figure 6:**
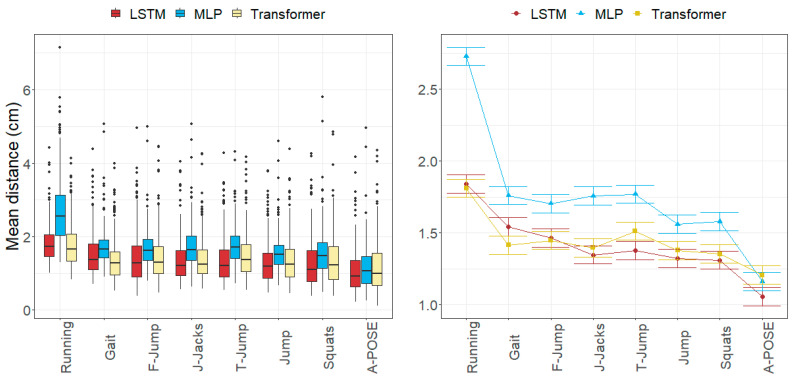
Errors in anatomical landmark positions depending on the movement and the model across all subjects in the test subset. (**Left**): observed distributions. (**Right**): marginal means of the LMM plus/minus their standard errors.

**Figure 7:**
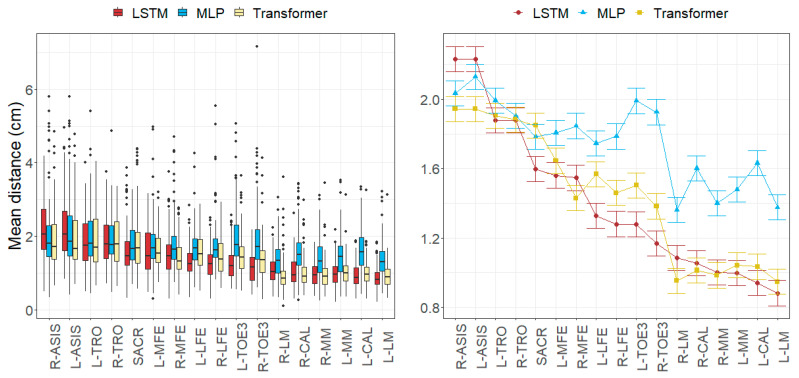
Errors in anatomical landmark positions depending on the anatomical landmark and the model across all subjects in the test subset. (**Left**): observed distributions. (**Right**): marginal means of the LMM plus/minus their standard errors.

**Figure 8:**
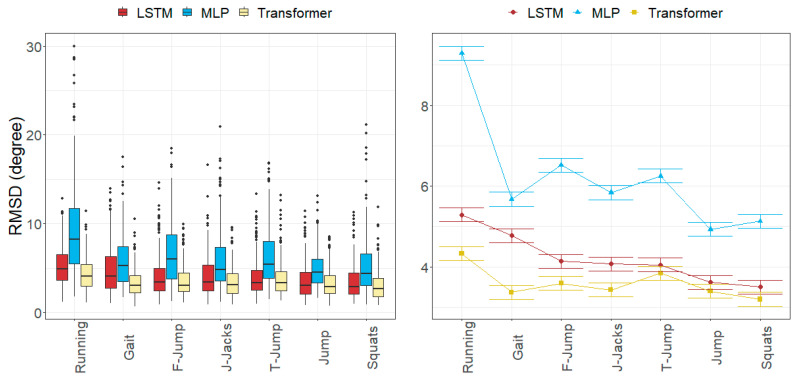
Errors in joint angles depending on the movement and the model across all subjects in the test subset. (**Left**): observed distributions. (**Right**): marginal means of the LMM plus/minus their standard errors.

**Figure 9:**
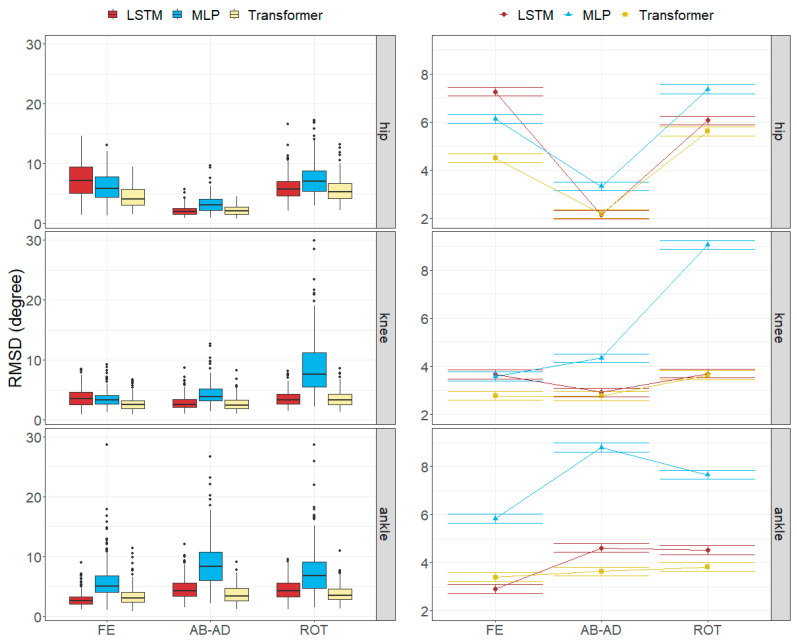
Errors in joint angles depending on the joint, rotation axis and the model across all subjects in the test subset. (**Left**): observed distributions. (**Right**): marginal means of the LMM plus/minus their standard errors.

Figure 6, Figure 7, Figure 8 and Figure 9 represent the distributions of the observed errors and their expected values according to the LMM, accounting for different factors and their interactions with the model. An advantage of the LSTM and the Transformer over the MLP model can be observed for both anatomical landmark position and joint angle errors, although the improvement is less than 1 cm and 3 degrees, respectively (Table 10). The performances of the LSTM and the Transformer models are similar, with a small advantage (<1 degree on average) for the Transformer in gait and running joint angles, mostly due to differences in hip flexion–extension.

## 4. Discussion

This study aimed to assess the suitability of using a marker augmentation model to convert the keypoints detected in images by standard pose estimation networks into anatomical landmarks, enabling the calculation of joint kinematics. Additionally, the study aimed to identify the factors that mainly affect their performance.

### 4.1. Size of the Joint Angle and Landmark Position Errors

To gain a comprehensive understanding of the results obtained, we reviewed studies that focus on comparing errors between markerless and marker-based photogrammetry MoCap systems and referred to research on the effect of marker placement to assess the size of errors in our results [58].

Markerless vs. marker-based studies typically reported errors in the positions of landmarks, joint centers, and joint angles along various axes (such as hip, knee, and ankle flexo-extension and hip abduction–adduction and rotation). In all cases, our comparisons were always performed with the lowest reported errors, such as those from studies using the highest-resolution pose estimators and the maximum number of cameras, or those reported under conditions similar to our work (e.g., studies involving sports clothing).

The anatomical landmark position errors of all the models in the reference A-pose were of the same order of magnitude as the intra-examiner position errors reported in work [58], indicating that our models and marker-based MoCap systems yielded similar levels of anatomical landmark position uncertainty.

We took the joint angle errors observed in gait movement as the reference values with which to compare the effects of marker position reported in work [58]. The magnitude of joint angle errors was similar to or lower than the uncertainty in joint rotations typically introduced by inter-examiner marker positioning errors in all three models, except for hip flexion–extension in the LSTM model (9.32 degrees vs. 5 degrees) and ankle flexion–extension in the MLP model (5.33 degrees vs. 3.3 degrees). 

We found several references reporting anatomical landmarks or joint position errors for gait movement [12,25,36,59], with the smallest average error being 1.22 cm [36]. The Transformer model was the only one that achieved comparable errors (1.42 cm). MLP and LSTM errors were of the same order of magnitude as or smaller than the errors in the rest of the studies (ranging between an average of 1.81 and 2.97 cm).

We could also compare the anatomical landmark position errors in running to those reported in [60]. LSTM and Transformer models exhibited smaller errors compared to the literature (1.84 vs. 2.32 cm), whereas the MLP model did not.

With respect to squats and jumps, we verified that the anatomical landmark position errors of all three models (each under 1.6 cm) improved upon those reported in the literature [25] (over 2.2 cm).

When considering joint angle errors, we observed that for gait movement, the errors for the Transformer model (average of 3.37 degrees) were lower than those found in the literature. Next, the performance of LSTM model was comparable to that reported in [25] (4.77 degrees vs. 4.76 degrees). The MLP model achieved joint angle errors of 5.68 degrees, slightly lower compared to those reported in other literature (ranging from 6.9 degrees and above) [12,49,59,61]. 

The joint angle errors in running for the Transformer model were the lowest found, with an average of 4.32 degrees, followed by the errors in LSTM (5.29 degrees) and those reported in [61] (6.26 degrees). The results of the MLP model (9.29 degrees) and [49] were far from the best achieved.

The comparison conducted for the squats and jumps led to similar conclusions. All three models improved the errors in ankle (equal to or under 4.17 degrees) and knee (equal to or under 3.4 degrees) flexo-extension, as well as hip abduction–adduction (equal to or under 3.1 degrees). However, only the Transformer model showed improvement in hip flexion–extension error.

### 4.2. Factors Influencing the Errors

The statistical analysis revealed that the anthropometric characteristics of the subjects did not significantly affect the errors in the positions of anatomical landmarks or joint angles. Therefore, all the variation that could be attributed to the physical characteristics of the subjects was fully captured by the model and did not have a significant influence on the errors.

Only half of the error variance could be explained by the considered factors, which included model, movement, and anatomical landmark for anatomical landmark position errors, and model, movement, joint, axis, and side for joint angle errors. The factors that most influenced the errors were the anatomical landmark and movement for anatomical landmark position errors, and model, rotation axis, and movement for joint angle errors. The interaction between joint and rotation axis was particularly relevant.

In general, anatomical landmark position errors were greater for the MLP model and smaller for the LSTM and Transformer models, depending on the movement. 

The anatomical landmark position errors reported for all the models followed a common pattern. Errors of the anatomical landmarks located on the pelvis (approximately 1.95 cm for ASIS, TRO, and SACR) were slightly greater than those of landmarks on the knees (LFE and MFE) and TOE3 (ranging from 1.43 to 1.80 cm). The smallest errors (ranging from 1.05 to 1.60 cm) were found in the anatomical landmarks in the ankle area (MM, LM, and CAL). Regarding anatomical landmark position errors per movement, the greatest errors were observed in running in all models (ranging from 1.81 to 2.73 cm), while the smallest errors were found in jumps and squats (ranging from 1.31 to 1.58 cm). These error patterns were consistent with those found in the literature. 

The joint angle errors in the Transformer model were generally the smallest for all the movements, while MLP provided the highest errors.

The axial rotation angle had the greatest error in all models, ranging from 3.64 to 9.06 degrees. Flexion–extension errors averaged 3.56 degrees for the Transformer and 4.62 degrees for LSTM, which were larger than the abduction–adduction errors, except for MLP (5.18 degrees). The abduction–adduction axis yielded the best results for the LSTM (3.22 degrees) and Transformer models (2.86 degrees).

Similar to anatomical landmark position errors, errors in the running movement were the largest for all models (9.29 degrees for MLP, 5.29 degrees for LSTM, and 4.32 degrees for Transformer). Conversely, joint angle errors were smallest in jump and squat movements (5.03 degrees for MLP, 3.55 degrees for LSTM, and 3.29 degrees for Transformer). 

Finally, we observed that flexion–extension angle errors at the ankles tended to be the lowest, averaging between 2.92 and 5.84 degrees, whereas the abduction–adduction angle error at the hips was the smallest, with errors ranging from 2.13 to 3.32 degrees. In the knee, rotation angles were commonly the worst estimated, with errors ranging between 3.64 and 9.06 degrees.

### 4.3. Other Considerations

The data used for this study were specifically designed to minimize the 3D reconstruction errors of keypoints in images. It is worth noting that 48 different points of view were generated per timeframe. Further research will focus on assessing the proposed models using a real setup with a specific number of cameras that provide an equivalent reconstruction error.

An interesting finding was that the proposed Transformer model exhibited similar errors for anatomical landmark position compared to the LSTM model, while demonstrating smaller errors for joint angles. 

The Transformer model, by performing self-attention for each patch of the input sequence in an N-to-N manner, outputs a set of context-enriched features, thus obtaining a global understanding of the problem [44]. This comprehensive understanding enables the model to effectively capture long-range dependencies and intricate relationships between keypoints in 3D space. By assigning varying degrees of importance to different patches based on their relevance to one another, the Transformer excels in discerning the underlying structure of the keypoints, ultimately leading to superior performance in understanding augmented sets of 3D anatomical landmarks and achieving lower angle errors.

As discussed, the Transformer model improved the anatomical landmark position errors of the LSTM model in f-jump, gait, and running movements, the ones that precisely involve the translation of the body in the anteroposterior axis.

The analysis of the anatomical landmark position and the joint angle errors indicated that the movement factor was particularly relevant. Our results revealed that errors in running were consistently 10% to 45% bigger than errors in other movements. Therefore, it may be reasonable to develop a model specifically tailored for running assessment.

## 5. Conclusions

An assessment of three deep learning models for marker augmentation was conducted, revealing distinct performance levels across each model. 

Through the testing and comparison across seven movements and three models (MLP, LSTM, Transformer), we identified the MLP model as the one which provides lower accuracy in terms of both anatomical landmark position errors and joint angle errors. The LSTM and Transformer models provided similar results, surpassing the Transformer model the LSTM model in the joint angle errors. While the joints angle errors in the LSTM model ranged from 3.5 to 5.29 degrees, in the Transformer model, they ranged from 3.19 to 4.32 degrees. The Transformer model might achieve a global understanding of the keypoints’ 3D relationships utilizing self-attention mechanisms. However, the selection of the model depends on the final application and the increase in accuracy is usually accompanied by an increase in model parameters. 

The anthropometric characteristics of the subjects had no significant impact on the errors associated with anatomical landmarks or joint angles, suggesting that the models’ performance is robust across different body types and sizes.

The analysis revealed that errors in running were consistently higher than in other movements, manifesting the influence of movement on the behavior of the models.

Anatomical landmarks were another factor that influenced the magnitude of errors. We observed that those placed on the pelvis are prone to having the biggest errors, whereas those on the ankle had the lowest.

Hip abduction–adduction and ankle flexion–extension angles were the best estimated at each joint for all models. Conversely, knee rotation angles were poorly estimated by all three models. 

This work introduces a new framework to the research community and is expected to contribute to the enhancement of markerless MoCap models.

## Figures and Tables

**Table 1:** Anthropometric characteristics of the dataset and training and test subsets.

Subset	Sex (Size)	Height (m) Mean (std)	Weight (kg) Mean (std)	Age (years) Mean (std)
Training	Female (*N* = 29)	1.64 (0.08)	70.89 (18.19)	38.21 (13.27)
	Male (*N* = 28)	1.78 (0.11)	79.77 (17.83)	37.04 (11.27)
	Total (*N* = 57)	1.71 (0.12)	75.25 (18.41)	37.63 (12.23)
Test	Female (*N* = 7)	1.58 (0.08)	55.57 (11.40)	34.86 (11.36)
	Male (*N* = 7)	1.75 (0.08)	78.54 (11.49)	31.14 (9.41)
	Total (*N* = 14)	1.67 (0.12)	67.06 (16.22)	33.00 (10.21)
Dataset	Total (*N* = 71)	1.70 (0.12)	73.64 (18.18)	36.72 (11.94)

**Table 2:** Errors (mean Euclidean distance in cm) for each anatomical landmark and movement across all subjects in the test subset with the MLP model.

Anatomical Landmark (AL)	A-Pose	Gait	F-Jump	J-Jacks	Jump	Running	Squats	T-Jump	Across Movement
L-ASIS	1.49	2.18	2.18	1.96	1.97	2.96	2.09	2.21	2.13
L-CAL	0.98	1.84	1.37	1.91	1.36	2.65	1.39	1.55	1.63
L-LFE	1.37	1.58	1.60	1.85	1.50	2.53	1.81	1.72	1.75
L-LM	0.82	1.39	1.27	1.60	1.24	2.21	1.08	1.42	1.38
L-MFE	0.99	1.85	1.70	1.91	1.59	2.93	1.80	1.69	1.81
L-MM	0.82	1.60	1.45	1.68	1.33	2.42	1.18	1.35	1.48
L-TRO	1.37	1.78	1.91	1.95	1.97	2.82	1.93	2.20	1.99
L-TOE3	1.19	2.05	1.90	1.96	1.65	3.44	1.51	2.22	1.99
R-ASIS	1.43	2.30	2.27	1.80	1.96	2.56	1.99	1.97	2.03
R-CAL	1.04	1.57	1.52	1.82	1.37	2.53	1.22	1.75	1.60
R-LFE	1.30	1.66	1.62	1.80	1.53	3.12	1.61	1.64	1.79
R-LM	0.77	1.40	1.51	1.43	1.11	2.09	1.15	1.44	1.36
R-MFE	1.37	1.96	1.64	1.68	1.55	3.27	1.56	1.74	1.85
R-MM	1.00	1.31	1.47	1.41	1.24	2.18	1.18	1.41	1.40
R-TRO	1.30	1.86	1.91	1.77	1.76	2.62	2.01	1.99	1.90
R-TOE3	1.26	2.07	1.64	1.63	1.53	3.75	1.47	2.05	1.93
SACR	1.27	1.53	2.03	1.71	1.88	2.28	1.86	1.72	1.78
Across AL	1.16	1.76	1.70	1.76	1.56	2.73	1.58	1.77	1.75

**Table 3:** Errors (mean Euclidean distance in cm) for each anatomical landmark and movement across all subjects in the test subset with the LSTM model.

Anatomical Landmark (AL)	A-Pose	Gait	F-Jump	J-Jacks	Jump	Running	Squats	T-Jump	Across Movement
L-ASIS	1.68	2.38	2.74	1.91	2.23	2.21	2.39	2.31	2.23
L-CAL	0.64	1.00	0.92	1.20	0.83	1.34	0.69	0.92	0.94
L-LFE	1.00	1.42	1.46	1.18	1.27	1.72	1.30	1.26	1.33
L-LM	0.63	0.90	0.82	0.78	0.80	1.43	0.86	0.84	0.88
L-MFE	0.95	2.06	1.64	1.43	1.26	2.41	1.33	1.39	1.56
L-MM	0.65	1.13	0.97	1.06	0.88	1.63	0.80	0.86	1.00
L-TRO	1.32	1.85	2.24	1.69	1.93	1.96	2.06	1.97	1.88
L-TOE3	1.03	1.48	1.37	1.31	1.03	1.90	0.91	1.19	1.28
R-ASIS	1.84	2.42	2.63	2.05	2.26	2.12	2.26	2.29	2.23
R-CAL	0.93	0.96	1.01	0.99	1.03	1.52	0.97	1.04	1.05
R-LFE	0.82	1.50	1.13	1.12	1.23	1.98	1.23	1.24	1.28
R-LM	0.88	1.19	0.90	0.98	1.01	1.78	0.96	0.98	1.08
R-MFE	1.10	1.94	1.40	1.59	1.24	2.57	1.18	1.37	1.55
R-MM	0.76	1.17	0.88	1.05	0.85	1.63	0.78	0.90	1.00
R-TRO	1.62	1.79	2.10	1.74	1.94	1.67	2.10	2.06	1.88
R-TOE3	0.84	1.41	0.99	1.26	1.03	1.79	0.82	1.20	1.17
SACR	1.29	1.60	1.75	1.55	1.66	1.66	1.62	1.63	1.60
Across AL	1.06	1.54	1.47	1.35	1.32	1.84	1.31	1.38	1.41

**Table 4:** Errors (mean Euclidean distance in cm) for each anatomical landmark and movement across all subjects in the test subset with the Transformer model.

Anatomical Landmark (AL)	A-Pose	Gait	F-Jump	J-Jacks	Jump	Running	Squats	T-Jump	Across Movement
L-ASIS	1.76	1.80	2.01	1.70	2.02	2.11	2.03	2.13	1.94
L-CAL	0.86	1.05	0.99	1.00	0.96	1.53	0.90	0.99	1.03
L-LFE	1.47	1.63	1.51	1.58	1.58	1.63	1.50	1.64	1.57
L-LM	0.74	0.85	0.85	0.97	0.92	1.43	0.89	0.95	0.95
L-MFE	1.56	1.73	1.66	1.72	1.61	1.69	1.54	1.66	1.65
L-MM	0.77	1.04	1.00	1.16	0.94	1.46	0.98	1.00	1.04
L-TRO	1.76	1.73	1.90	1.63	2.03	2.00	2.08	2.13	1.91
L-TOE3	1.13	1.52	1.59	1.51	1.25	2.22	1.16	1.65	1.50
R-ASIS	1.73	1.83	2.02	1.80	2.07	1.93	1.97	2.19	1.94
R-CAL	0.67	0.96	0.93	1.06	0.83	1.75	0.87	1.04	1.01
R-LFE	1.20	1.42	1.58	1.39	1.33	1.89	1.40	1.46	1.46
R-LM	0.55	0.98	0.91	0.93	0.73	1.84	0.78	0.91	0.95
R-MFE	1.30	1.44	1.51	1.44	1.33	1.61	1.38	1.43	1.43
R-MM	0.72	0.95	0.95	1.00	0.83	1.60	0.86	0.96	0.98
R-TRO	1.72	1.81	1.90	1.68	1.95	1.94	1.97	2.09	1.88
R-TOE3	0.81	1.55	1.41	1.42	1.16	2.31	0.93	1.48	1.38
SACR	1.78	1.77	1.92	1.76	1.92	1.84	1.78	2.02	1.85
Across AL	1.21	1.42	1.45	1.40	1.38	1.81	1.35	1.51	1.44

**Table 5:** Average of RMSD (degrees) for each joint, axis, and movement across all subjects in the test subset with the MLP model.

Movement	Hip			Knee			Ankle			Across Joint and Axis
	FE	AB-AD	ROT	FE	AB-AD	ROT	FE	AB-AD	ROT	
Running	7.39	5.21	9.04	4.67	6.59	15.35	10.1	12.86	12.44	9.29
Gait	5.13	3.13	6.71	3.55	3.64	8.08	5.33	8.73	6.77	5.68
F-Jump	7.62	2.97	6.24	3.49	3.94	10.58	5.82	9.01	8.99	6.52
J-Jacks	5.25	2.52	7.96	3.89	3.77	7.73	7.06	7.74	6.63	5.84
T-Jump	5.01	3.48	9.51	3.07	4.7	8.68	5.31	9.02	7.45	6.25
Jump	6.08	2.8	6.06	3.06	3.85	5.93	4.17	6.92	5.48	4.93
Squats	6.4	3.1	6	3.37	3.9	7.09	3.09	7.3	5.89	5.13
Across movement	6.12	3.32	7.36	3.59	4.34	9.06	5.84	8.8	7.66	6.23

**Table 6:** Average of RMSD (degrees) for each joint, axis, and movement across all subjects in the test subset with the LSTM model.

Movement	Hip			Knee			Ankle			Across Joint and Axis
	FE	AB-AD	ROT	FE	AB-AD	ROT	FE	AB-AD	ROT	
Running	7.62	2.9	6.52	4.54	4.12	5.52	4.3	5.63	6.44	5.29
Gait	9.32	2.04	6.53	4.29	2.96	3.73	2.93	5.64	5.48	4.77
F-Jump	8.63	1.96	5.5	3.53	2.99	3.89	2.2	4.68	3.84	4.14
J-Jacks	5.13	2.1	7.24	2.82	2.69	3.2	3.79	4.76	4.93	4.07
T-Jump	7.15	2.31	6.65	3.24	2.67	3.37	2.92	4	4.08	4.04
Jump	6.82	1.78	5.2	3.4	2.42	3.19	2.29	3.94	3.5	3.61
Squats	6.17	1.86	4.78	3.88	2.6	3.03	1.99	3.68	3.47	3.5
Across movement	7.26	2.13	6.06	3.67	2.92	3.7	2.92	4.62	4.53	4.2

**Table 7:** Average of RMSD (degrees) for each joint, axis, and movement across all subjects in the test subset with the Transformer model.

Movement	Hip			Knee			Ankle			Across Joint and Axis
	FE	AB-AD	ROT	FE	AB-AD	ROT	FE	AB-AD	ROT	
Running	4.94	2.63	5.34	3.47	3.23	4.31	5.7	4.98	4.33	4.32
Gait	3.99	1.91	4.78	2.92	2.2	3.33	3.49	4.17	3.52	3.37
F-Jump	5.26	2.16	5.66	2.72	2.74	3.8	3.06	3.27	3.64	3.59
J-Jacks	3.52	2.03	5.6	2.21	2.41	3.57	3.73	3.97	3.84	3.43
T-Jump	4.91	2.43	7.13	2.76	2.98	3.84	3.2	3.31	4.02	3.84
Jump	4.78	1.95	5.67	2.6	2.83	3.34	2.63	3.16	3.55	3.39
Squats	4.07	2.07	5.09	2.74	2.99	3.3	1.99	2.63	3.85	3.19
Across movement	4.49	2.17	5.61	2.78	2.77	3.64	3.4	3.64	3.82	3.59

**Table 8:** ANOVA table for anatomical landmark position errors (SS: sum of squares, MS: mean squares, DoF: degrees of freedom).

	SS	MS	DoF
Model	137.88	68.94	2
Movement	381.41	54.49	7
Anatomical landmark	617.89	38.62	16
Model:Movement	78.22	5.59	14
Model:Anatomical landmark	88.55	2.77	32

Conditional R^2^ = 0.509, Marginal R^2^ = 0.431.

**Table 9:** ANOVA table for joint angle errors (SS: sum of squares, MS: mean squares, DoF: degrees of freedom).

	SS	MS	DoF
Model	6745.12	3372.56	2
Movement	2828.23	471.37	6
Joint	1032.79	516.40	2
Axis	3186.66	1593.33	2
Side	67.53	67.53	1
Model:Movement	1233.55	102.80	4
Model:Joint	1454.16	363.54	12
Model:Axis	1195.82	298.95	4
Joint:Axis	4614.30	1153.58	4
Model:Joint:Axis	1593.14	199.14	8

Conditional R^2^ = 0.538, Marginal R^2^ = 0.516.

**Table 10:** Marginal errors for the different models according to the fitted LMMs.

	MLP	LSTM	Transformer
Anatomical landmark distance (cm)	1.75	1.41	1.44
RMSD (degrees)	6.23	4.20	3.59

## Data Availability

The “Human tracking dataset of 3D anatomical landmarks and pose keypoints” is available at https://data.mendeley.com/datasets/493s6f753v/2, accessed on 31 January 2024. Neural network architecture, data pre-processing, and training hyperparameters are described within the article. Appendix A details the content in the test subset and details the anatomical landmarks taken from the dataset.

## Appendix A

The following table relates the denomination used in this work to the names of the anatomical landmarks denoted in the dataset used.

**Anatomical Landmarks**	**Name in Dataset**
L-ASIS	Lt ASIS
L-CAL	Lt Calcaneous Post
L-LFE	Lt Femoral Lateral Epicn
L-LM	Lt Lateral Malleolus
L-MFE	Lt Femoral Medial Epicn
L-MM	Lt Medial Malleolus
L-TRO	Lt Trochanterion
L-TOE3	Lt Digit II
R-ASIS	Rt ASIS
R-CAL	Rt Calcaneous Post
R-LFE	Rt Femoral Lateral Epicn
R-LM	Rt Lateral Malleolus
R-MFE	Rt Femoral Medial Epicn
R-MM	Rt Medial Malleolus
R- TRO	Rt Trochanterion
R-TOE3	Rt Digit II
SACR	This point is midpoint between Lt PSIS and Rt PSIS

The subjects in the test set were the following (the rest remaining in the training set): TDB_004_F, TDB_011_M, TDB_028_M, TDB_032_F, TDB_035_F, TDB_037_M, TDB_038_M, TDB_041_F, TDB_042_F, TDB_049_F, TDB_053_M, TDB_055_M, TDB_061_M, and TDB_071_F.
